# Towards a common framework for assessing the activity and associations of groups who sexually abuse children

**DOI:** 10.1080/13552600.2013.791730

**Published:** 2013-05-01

**Authors:** Ella Cockbain, Helen Brayley, Joe Sullivan

**Affiliations:** 1 Department of Security and Crime Science, University College London, London, UK; 2 Behavioural Analysis Unit, Child Exploitation and Online Protection Centre (CEOP), Part of the Serious Organised Crime Agency (SOCA), London, UK

**Keywords:** Criminal organisation, child sexual abuse, child sexual exploitation, co-offending, offender interviews

## Abstract

Extensive social psychological research emphasises the importance of groups in shaping individuals’ thoughts and actions. Within the child sexual abuse (CSA) literature criminal organisation has been largely overlooked, with some key exceptions. This research was a novel collaboration between academia and the UK's Child Exploitation and Online Protection Centre (CEOP). Starting from the premise that the group is, in itself, a form of social situation affecting abuse, it offers the first systematic situational analysis of CSA groups. In-depth behavioural data from a small sample of convicted CSA group-offenders (n = 3) were analysed qualitatively to identify factors and processes underpinning CSA groups’ activities and associations: group formation, evolution, identity and resources. The results emphasise CSA groups’ variability, fluidity and dynamism. The foundations of a general framework are proposed for researching and assessing CSA groups and designing effective interventions. It is hoped that this work will stimulate discussion and development in this long-neglected area of CSA, helping to build a coherent knowledge-base.

## Introduction

Humans’ thoughts, beliefs and actions are influenced heavily by “the power of the social situation” (Banaji, 2001, p. 15). A large proportion of crime involves groups, which “has fundamental but often unacknowledged implications for crime analysis and criminal justice” (Van Mastrigt & Farrington, 2009, p. 553). While crimes need not feature multiple offenders to be “social”, embedded as they are within overarching social structures, when they do this social dimension is particularly pronounced. There is an extensive body of theory and empirical evidence attesting to the power of the group in influencing members’ thoughts and actions. Processes such as conformity (Sherif, 1935), obedience (Milgram, 1963), imitation (Bandura, Ross, & Ross, 1961), deindividuation (Festinger, Pepitone & Newcomb 1952) and diffusion of responsibility (Bandura, Underwood, & Fromson, 1975) have helped explain group influences on antisocial conduct within diverse experimental and real-world contexts.

In the 1970s, Cohen (1977, p. 11) observed that a lack of research interest in criminal organisation had left major knowledge-gaps around the “specific social arrangements for the accomplishment of crime”, such as recruitment. To a lesser extent, similar could be said today, with the notable exceptions of a flurry of interest in the definition and traits of organised crime groups from the mid-1990s (see Finckenauer, 2005; Paoli, 2002) and an important but fragmented literature on direct collaboration in crime (co-offending) (see Andresen & Felson, 2010a, 2010b; Hindelang, 1971, 1976; Reiss, 1988; Van Mastrigt & Farrington, 2009). While the representativeness of co-offending research is limited by its skewed focus on the United States, juvenile offenders and so-called “volume crime”, co-offending has been found repeatedly to be a common feature of crime (Andresen & Felson, 2010a; Reiss, 1988). Offenders are more likely to commit both co-offences and solo-offences than just one or the other, and those who co-offend commit more offences (Hindelang, 1971, 1976; Reiss, 1988): co-offending may, in itself, be criminogenic (Andreson & Felson, 2010b). Groups are thought to generate unrivalled opportunities for crime, enabling direct collaboration and the spread of information and other commodities (Andreson & Felson, 2010b; Clarke, 1992).

In general, however, criminology has neglected groups, focusing primarily on individual disposition or, in the case of situational theories, individual decision-making (Andreson & Felson, 2010b). Although the crude distinction between dispositional and situational approaches is explained here, all behaviour is the result of the interaction between individual (disposition) and environment (situation) (Mischel, 1968). As such, the two are best seen as complementary parts of crime's multi-dimensional aetiology (Clarke, 1995). Dispositional research focuses on criminality and its provenance, considering psychological, socioeconomic and biological processes by which people become offenders (Clarke, 1997). In contrast, situational research focuses on offender decision-making, seeking to identify those physical and social aspects of immediate situations that affect perceptions of and responses to offending opportunities (Clarke, 1983; Cohen & Felson, 1979; Cornish & Clarke, 1986). Attempts to identify (and tackle) situational drivers of crime, it must be emphasised, do not exculpate offenders.

Within the rich literature on child sexual abuse (CSA), the majority of work has sought to identify traits distinguishing offenders from the wider population and to inform, thereby, effective treatment (Marshall, Serran, & Marshall, 2006; Wortley & Smallbone, 2012). Far less attention has been paid to the behaviour of these offenders. In recent years there has been increased interest in situational approaches to CSA (Wortley & Smallbone, 2006), against a backdrop of growing evidence that situational approaches are efficacious in tackling not only “volume crime”, but also serious and organised offences (Clarke, 1997). Situational approaches present a valuable opportunity to address the complex social issue that is CSA from a fresh angle (Kaufman, Mosher, Carter, & Estes, 2006; Taylor & Quayle, 2006). Most situational work in this field to date has focused upon individual offenders’ *modi operandi* (for a review, see LeClerc, Proulx, & Beauregard, 2009). Situational approaches, like dispositional ones, have paid little systematic attention to understanding the activities and associations of CSA groups.

Until recently, those exceptional studies that measured group involvement in CSA found low levels. What is thought to be the largest such study to date found that of the 182 convicted CSA offenders questioned, only 9% (*n* = 16) had been knowingly involved with other CSA offenders pre-arrest and only 4% (*n* = 7) had co-offended (Smallbone & Wortley, 2000). Sex crimes against adults are also primarily a solo occupation, with the exception of very specific contexts such as within gangs (Batchelor, 2009; Wood, 2005), college fraternities (Sanday, 1990), or during wars (Wood, 2006). In the first large-scale British study of co-offending rates across a wide range of crime types, Van Mastrigt & Farrington (2009) found that sexual offences (*n* = 213) had one of the lowest co-offending rates at only 4.7%. Given the enduring taboos against sexual offences, in particular against children, such results are not entirely surprising.

Recent developments, however, question the primacy of the solo CSA offender. First, at a transnational level, the internet's proliferation has created new opportunities for those with a sexual interest in children to seek out like-minded individuals. Fears of stigmatisation and apprehension can be alleviated by the internet's accessibility, isolation and anonymity, or the illusion thereof (Carnes, Griffin, & Delmonico, 2001; Wortley & Smallbone, 2012). The internet can facilitate exchanges with an instrumental (e.g. image transfer) or expressive (e.g. mutual support) function, the latter of which may have a particular effect on offenders’ cognitions (see, for example, Durkin & Bryant, 1999; O'Halloran & Quayle, 2010; Taylor & Quayle, 2003). Secondly, in the United Kingdom there has been a rapid growth of interest in child sexual exploitation (CSE), a poorly delineated subset of CSA, involving multiple offenders (see, for example, Cockbain, 2013). A national threat assessment of ‘localised grooming’ (broadly defined as CSE perpetrated by adults in an offline, extra-familial, non-professional context) found that at least 31% (N = 734) of the suspected offenders acted in groups (CEOP, 2011). Since then, ‘group- and gang-associated CSE’ has become a UK child protection priority (CEOP, 2011) and been the subject of another major national study (OCCE, 2012). Unfortunately, what constitutes group association is never clarified: is it, for example, co-offending only, or does offender networking suffice? Moreover, as gangs are, by nature, groups, the distinction between the two is misleading.

Starting from the premise that group involvement is an integral part of the crime environment (Andreson & Felson, 2010b), in this paper CSA groups are explored from a situational perspective. In line with the situational approach, the focus is upon deconstructing “the group” as an immediate social environment that directly influences crime commission. The aim here was not to analyse the behaviour of a particular group, or even group activity in a particular form of CSA, but rather to begin to establish distinct dimensions against which all CSA groups can be assessed; for example, the mechanisms by which they form. To this end, extensive interview material from a small sample of group-associated CSA offenders (*n* = 3) was subject to in-depth analysis to test the utility of this approach in improving understanding of CSA groups. The results are by no means conclusive, but rather represent the first steps towards constructing a framework against which these and other situational dimensions to CSA groups could be assessed in a systematic and replicable manner. Such a framework could facilitate cross-study comparisons and the development of a coherent literature around this neglected aspect to CSA. From a practical perspective, this could guide resource allocation and inform counterstrategies that could inhibit the formation of groups and disrupt their activities.

In the absence of a consensual definition of groups, let alone crime groups, a working definition of a CSA group was required. Contrary to popular stereotypes of stable, fixed and hierarchical criminal groups, most co-offending groups are more like loose federations (Reiss, 1988) that may converge for the commission of a single crime and disband thereafter (Brantingham & Brantingham, 2008). Consequently, a broad, inclusive definition was favoured that accommodates both temporary and more permanent groups and does not require physical collocation, which would exclude online CSA groups. A CSA group is defined, therefore, as “a set of two or more connected child sex offenders, within which each individual has committed a child sex offence with at least one other group member”. The requirement that each member have co-offended with another differentiates the CSA group from a network of interconnected, but ultimately solo-offending, CSA perpetrators. Its inclusion means that this definition covers the two critical dimensions of criminal organisation: structures of association and of activity (Cohen, 1977). Indeed, shared activity involves task interdependence (Lewin, 1951), a core distinguishing characteristic of groups. However, the definition does not equate groups with mere co-offending constellations alone, recognising that CSA groups may, in addition, feature shared associations, structure, identity and social interdependence (Lewin, 1951).

## Method

### Data

The research data were provided by the Child Exploitation and Online Protection Centre (CEOP), part of the UK's Serious Organised Crime Agency (SOCA). As part of their ongoing research and training programme, CEOP's Behavioural Analysis Unit conducts interviews with diverse convicted CSA offenders: to date, 24 such individuals have been interviewed over the past decade. While the interview script did not feature group-specific questions, subjects had the opportunity to discuss their group involvement when answering questions about offending behaviour. To filter the archives inclusion criteria were developed, as shown in Table I. In brief, however, subjects were all adult males involved in co-offences and convicted in Britain, whose interviews were extensive enough to support in-depth analysis.

**Table I.** Criteria for inclusion in the study

Primary filtering function	Criteria
Relevance to the research question	• Subject involved in at least one child sex-offending group • Subject committed contact sex offences against a child(ren) • Subject demonstrated pattern of offending (multiple offences/multiple occasions)
Sufficient volume of data	• Subject interviewed on two or more occasions • Subject interviewed for at least 60 minutes per session • Subject interviewed for a minimum total of 3 hours
Focus and consistency	• Subject is male • Subject was an adult at time of offences in question • Subject is a British national convicted under UK law • When interviewed, the subject was in prison or under licence for child sexual offences

The majority of the 24 potential subjects were solo offenders only, and as such not suitable for this study; a few others were excluded due to age, gender or a non-UK conviction. Only three subjects met all 10 criteria, and together their offending histories span six decades, from the 1950s to the 2000s. Table II has brief descriptions of each: here, as throughout, names and identifiers are changed to protect subjects’ anonymity.

**Table II.** Description of subjects

Subject	Description
Simon	A 78-year-old white male, on licence for possessing indecent images of children. He was part of various different groups involved in contact child sex abuse, producing and distributing indecent images and support and advocacy for those with a sexual interest in children. Simon professes an exclusive sexual interest in boys aged 8–16 years, despite continuing sexual relations with one victim throughout his late teens and into early adulthood. Many of Simon's contact sexual offences took place while photographing children, often outside the United Kingdom. Prior to his arrest Simon lived alone, and he has never had an adult sexual relationship. He has been aware of his sexual attraction to children since his late teens
Clive	An 85-year-old white male, in prison for contact offences against his stepchildren. He is generally considered the leader of a violent group, which sexually exploited vulnerable boys. He may also have been involved in smaller, more impromptu groups and has a long history of solo offences. Clive's sexual interest spans both adults and children, male and female, although his preference is for pubescent and early post-pubescent boys. Clive began to be sexually interested in children in his teens and has committed both familial and non-familial contact offences over a 30-year period, in the United Kingdom and while abroad on military service. Clive has had several failed relationships with adult women, including the one whose children he abused
Les	A 35-year-old white male, in prison for contact offences and child pornography offences. He abused his own stepchildren and distributed the indecent images online. He also collected other indecent images via the internet. He was part of several online groups of varying sophistication, including one of the most prolific rings ever uncovered. Les became aware of his sexual interest in children in his late teens and describes struggling to control it for several years. He has been in several serious relationships with women and has one child of his own. His first experiences of contact offending were with the children of his now ex-wife. Due to the nature of online offending groups, Les has been involved in offence patterns that span multiple jurisdictions: his own contact offending, however, was concentrated in the United Kingdom

While all three subjects were involved in CSA groups, each also had a history of solo offending, as is common for co-offenders in general (Reiss, 1988). Sometimes a solo offence, such as the creation of child abuse images, created the substantive basis for a co-offence, such as the sharing of this material online. Because the study's aim was to deconstruct CSA groups in terms of their activity and associations, comparative analysis of the subjects’ solo and co-offences was not deemed relevant. Nonetheless, this may be a very fruitful avenue in future, in particular once a systematic approach to assessing group-based CSA has been formalised, building hopefully on this preliminary work.

### Ethics

All interviews were conducted by the third author (Joe Sullivan) in full compliance with CEOP's ethical review process and with prison access granted by the National Offender Management System. Subjects gave informed consent to participation, audiovisual recording, archival of these records and their use in future (non-specified) research in the interests of harm reduction. CEOP's research panel approved the use of these secondary data in this particular study prior to its commencement. All names and possible identifiers have been changed in order to protect subjects’ anonymity. The interviews were reviewed and transcribed in a secure office on CEOP's premises, where they are stored in accordance with the Data Protection Act 1998. All authors were security cleared to a high level.

### Analysis

This study's aim was to identify immediate situational processes and factors shaping the associations and activities of CSA groups. Sampling difficulties are relatively common when dealing with hard-to-reach populations such as CSA offenders and, although it would have been preferable to include more subjects, the benefit of the small sample (*n* = 3) was that it allowed detailed analysis of a problem that had not been addressed previously in this manner. A qualitative approach was preferred due to the extensive behavioural data required in order to deconstruct this complex phenomenon. The method of choice was thematic analysis, a flexible, adaptive method with a preference for small sample sizes. The analysis followed Braun and Clarke's (2006) six-stage model: familiarisation with data; generation of initial codes; search for themes; review of themes; definition and naming of themes; and production of the final report. This approach requires that three key parameters be set, the choices for which are summarised in Table III.

**Table III.** Key parameters for the thematic analysis

Key parameters	Option chosen	Reason for option choice
Theoretical framework	Situational theory	To redress knowledge gaps around situational processes and factors affecting CSA groups
Mode of analysis	Inductive (“bottom-up”)	To enable themes and subthemes to be generated from the data (Frith & Gleeson, 2004) rather than fitting the data to predetermined ideas (“top-down” analysis)
“Level” of analysis	Semantic	To address the explicit content to the interviews, rather than the way in which it was expressed (Boyatzis, 1998)

The first and second authors (Ella Cockbain and Helen Brayley) transcribed the interviews in full, entered the verbatim transcripts into the NVivo software package and analysed them using the six-stage model set out above. While coding, they strove to balance the need for focus and clarity with that of avoiding imposing assumptions and preconceptions onto the data. New nodes were created as required and material coded to an expanding set of nodes. Intercoder reliability was measured for one interview using Cohen's kappa (Lombard, Snyder-Duch, & Bracken, 2002): at 0.83 it was above the minimal requirement of 0.7 (Neuendorf, 2002). The few inconsistencies were addressed and the coding adjusted accordingly. A broad set of nodes was thus generated, providing the basis for developing and refining themes. For this, nodes were reviewed, clustered and then filtered to leave only those most relevant to the research question. Four themes were generated in this manner, each with a number of component subthemes.

## Results and implications

**Figure 1.**
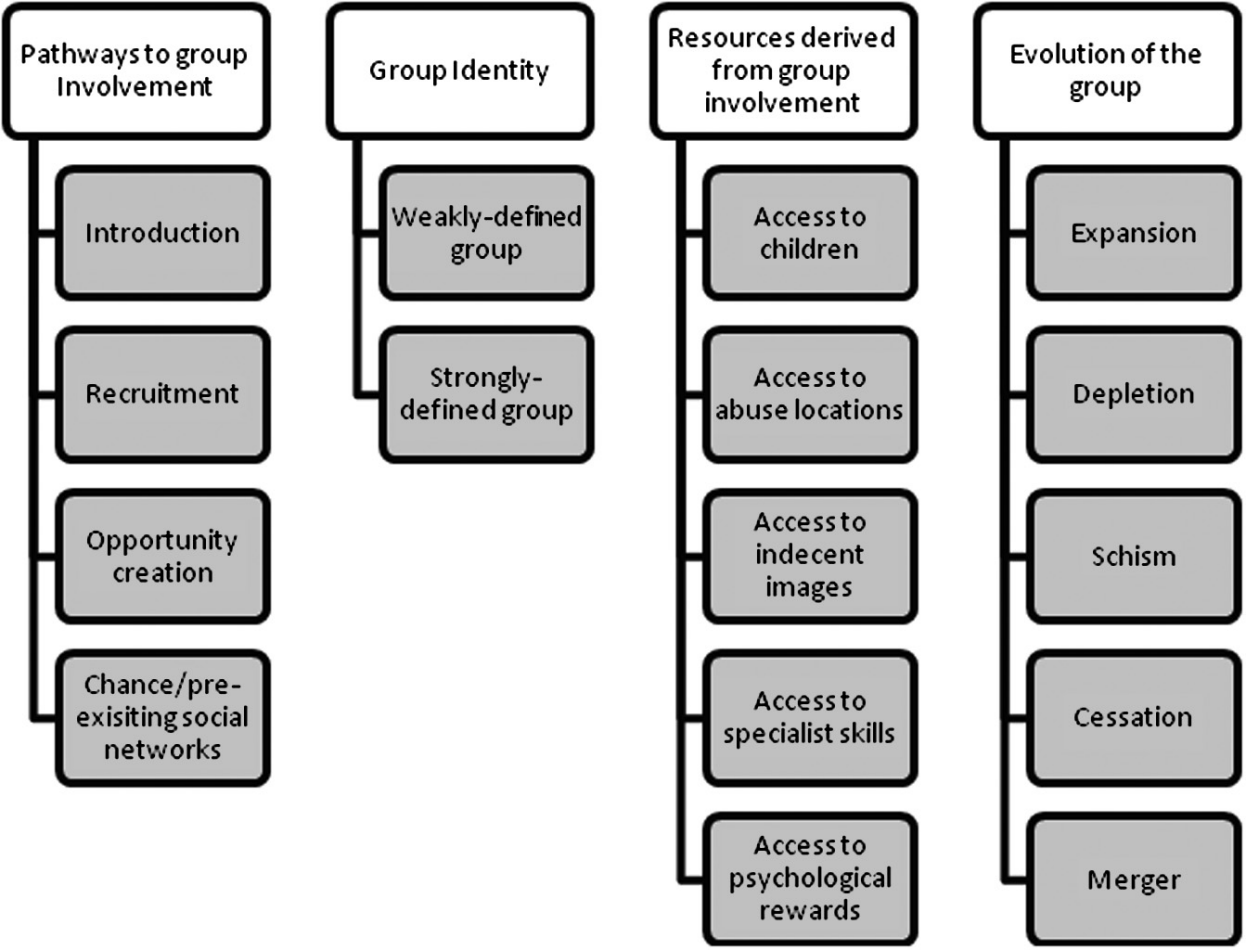
Themes and subthemes.

Each subject was involved in multiple groups, often at the same time, which varied greatly in their duration—from a matter of minutes to many weeks, months or even years. This underlines the multiplicity, fluidity and dynamism of group affiliations in CSA, something common to research into other forms of criminal collaboration (Reiss, 1988; Weerman, 2003). The subjects’ concurrent affiliations to several groups indicate that, for these groups at least, no member exclusivity was required. Figure 1 shows the four key themes and associated subthemes identified in the analysis of these diverse groups: each relates to a different aspect of group activity or association.

Each theme applied to all three subjects: indeed, it is suggested that most, if not all, CSA groups could be assessed along these themes. Within each, however, the subthemes relate to specific manifestations of the themes; for example, four different pathways to recruitment were identified. In some cases, subthemes are mutually exclusive alternatives (e.g. for group identity), whereas in others they may be complements (e.g. for group resources). No claim is made to have compiled the definitive, comprehensive framework for assessing CSA groups: neither the themes nor their subthemes are exhaustive. These are simply the first pieces, as it were, in a much bigger jigsaw puzzle that comprises group involvement in CSA.

### Pathways to group involvement

There was evidence of four distinct ways in which individuals became involved in CSA groups, each either taken actively or observed and discussed by these subjects.

*Through introduction.* A group can form, or expand, when offenders are introduced to one another by mutual contacts in their wider networks of association. Simon became involved with another travelling CSA offender in this way: the pair was then introduced, via a further contact, to a professional photographer who doubled as a producer of indecent images and procurer of children for contact abuse.

We had an introduction [letter] to someone he knew who was a photographer. [Simon]

Introductions were equally important online. Les, for example, was reluctant to engage when approached by a stranger, only reconsidering after a mutual acquaintance intervened.

I didn't know this guy from Adam and it wasn't until I went back online at a later date that someone that I did know said to me … he's alright, I know him. [Les]

Introductions may be a popular route into CSA groups due to the association with increased safety: mutual contacts acting as vouchsafes reduce the risks of inappropriately “outing” oneself as sexually interested in children and of law enforcement infiltration.

*Through recruitment.* Pre-existing groups or interested individuals may recruit others actively, providing a ready route into group involvement. Recruitment can be targeted or more general: Les, for example, was singled out as a producer of indecent images and asked to join a secretive but prolific online group, one of the most notorious to date:

All of a sudden I'm very popular and everyone's mad keen to say hello. [Les]

[T]hat was the only reason that they invited me to join the group. Otherwise, because I was so green … I would never have stood a chance. [Les]

Meanwhile, Simon was recruited by members of a CSA group that also advocated on behalf of those with a sexual interest in children who attended his trial, which had generated considerable publicity as one of the first prosecutions under new child pornography legislation.

There were two fellows turned up to my trial who I knew nothing about at the time … and over the following years we became very good friends. [Simon]

*Through opportunity creation.* Offenders may seek out one another actively, either by speculatively visiting places with reputations for sexual deviance or targeting specific locations, on- or offline, using privileged knowledge gained through their network of contacts. These approaches amount to the deliberate creation of opportunities for CSA group involvement, as opposed to merely capitalising on opportunities that present themselves. Clive highlighted several places where interested offenders could find like-minded individuals:

You can be in a toilet … hear them outside, you go into cafés you hear it, you go into pubs you hear it. I can name you … one, two, three, four, five, six, seven areas of Leeds. [Clive]

There is an online parallel to this: Les met his future group-mates by trawling indecent image sites:

I met SugarLove through this sort of bulletin board and we became quite friendly, chatting and talking. [Les]

Initially an observer, he gradually engaged with others in a more active manner:

I was what you call a lurker for long time, just sort of sat watching things … until I plucked up the courage to post myself. [Les]

*Through chance meetings and pre-existing social networks.* Groups can form through chance meetings in apparently random locations or arise within pre-existing social networks. Simon, for example, witnessed a CSA group form in this way, although he did not join in himself, when his colleagues became involved in sexually abusing children at work. From this data set, it remained unclear how offenders establish their shared sexual interest safely and reliably in such environments without obvious cues: perhaps subtle probes are used to test the water, rather than declaring interest or intent openly. This could mitigate the risks of negative responses, such as stigmatisation, attack or disclosure to police. Indeed, Les realised the danger of not concealing his sexual preferences after lending a colleague his “barely legal” magazine in which the centrefold featured readers’ photos of minors:

I once had a comment off a mate about the contents of the centre pages. After that I removed all the centre pages from those magazines so nobody got to see them. [Les]

These particular offenders formed no CSA groups, defined as involving co-offending, through prison, although Clive in particular referred to extensive networking on sex offender wings, sharing information about victims and *modi operandi.*

### Sense of group identity

Group identity can be an internal or an external construct. The former arises from *inside* a group, through members’ shared codes and values, for example. The latter is projected *upon* a group through the way outsiders react or refer to a set of individuals. The two can diverge greatly in the level of cohesion and identity they involve, as demonstrated by Clive's irritation that the group with whom he was tried was portrayed in the media as a clear-cut, regular circle of *n* offenders. This was at variance, he claimed, with its true identity. Similarly, Les objected to how his case was covered in the media as involving Group X, when in fact members of an entirely different group had been arrested:

… the police didn't arrest hardly anyone out of that group, it was the other one. [Les]

The subthemes below are based on examples of internally constructed identity, evident in the subjects’ interviews, although the distinction may be equally applicable to externally constructed identity. They represent two ends of what the authors posit is a spectrum running from casual, ill-defined, loose linkages to clear-cut groups with strong markers of membership.

*Weakly-defined groups.* Here, members do not see themselves as a group in any formal sense. Such groups may be more likely to form opportunistically: individual offenders converge temporarily in pursuit of a shared goal, such as image-transfer, and separate on completion of the task. Such short-term collectivities are unlikely to share a name, codes or lasting goals. It was difficult to identify cases of the absence of an identity in the transcripts, especially as subjects were not questioned directly about their groups. Simon, however, downplayed ties to one group by describing members as “acquaintances…rather than friends”.

In theory, CSA offenders as a whole could be viewed as a group, although not under the definition used here with its co-offending requirement. The notion that all CSA offenders are somehow alike was rejected by the subjects, all of whom, instead, emphasised the distinction between “good” and “bad” CSA offenders:

Certain people said, ‘They're the same bloody mob as you'. And I said, ‘No they ain't'. I said, ‘I don't have to go behind a fucking vicar's cloth. [Clive]

They hear the word ‘paedophile’ and think, ‘This is a chap who's going to be raping my three-year old daughter … there's just no way I'd have the slightest inclination. [Simon]

*Clearly-defined groups.* Here, groups share a name, fixed membership, terminology or other markers of a common identity. Simon was involved in a clearly identified group of so-called “intellectual paedophiles”, who engaged in advocacy as well as abuse. Meanwhile, Les was involved with several clearly identified groups online:

[We were] known as SKIF … like the initial of each of our nicknames. [Les]

### Resources derived from group involvement

Motivations to form or join groups can differ greatly from the actual benefits of group involvement: while the data gave little insight as to the former, the subjects revealed much about the latter. A major advantage of group involvement seems to be access to resources, of which five categories were identified here, including those critical to the crime-commission process. Depending on the individual offender, group involvement may be a prerequisite for accessing such resources, or a way to accrue them more quickly, with less risk or in greater quantities.

*Access to children.* Group involvement can give subjects access to more children, including those to whom they are less attached and from whom there is less risk of disclosure, thus enabling abuse to escalate. Before joining a group of seasoned offenders Simon's CSA was limited to photographing, and occasionally groping, boys he met opportunistically. Once in the group, however, he had ready access to his co-offenders’ so-called “ménage” of longstanding victims:

He wasn't only photographing him, he was screwing him fairly regularly and most of the other boys as well: so the boys were very experienced … they were perfectly happy. [Simon]

Like Les, Clive already abused his own stepchildren by himself, but through his groups he gained access to a wide range of other children, abducted or coerced into exploitation by various members. The CSA in which they engaged was largely non-commercial, although there were clear instances of payments to a third-party facilitator and/or to the victim:

That person who supplies the good is paid for supplying the goods … and then that young person has to be paid …in four hours she's been abused by about 10 or 12 people. She only gets one payment, she doesn't get payment for him, him, him, him. [Clive]

In CSA with a commercial element such as this, group involvement enables offenders to pool their funds: what might be too low a “fee” from one offender alone may become acceptable if matched by others:

You'll put so much in the pot for that young person who's got to give you entertainment for the night. [Clive]

Well, one's that way, one's having that way, one's down that way. He gets it three ways … and they may only pay about ten quid and they've got him all night. [Clive]

*Access to abuse locations.* These subjects described diverse types of abuse location, varying in their comfort, convenience and security. Often, group involvement gave offenders knowledge of and access to desirable locations. Simon, for example, was taken to isolated rural spots of which he, as a foreigner and outsider, would never have been aware:

We would go off each day in the minibus to some convenient place. It might be a nice sort of secluded castle, it might be a wood. [Simon]

In cases such as this, access to abuse locations went hand-in-hand with access to children, who were procured by a group member for the others. Clive described invitation-only parties hosted by a group member in his own home:

Eight or nine adults come to his flat … and he'd go, ‘Come in the bedroom'. And he's got the children there starkers. [Clive]

He also mentioned so-called “syndicates”: highly organised enterprises offering members an “all-inclusive” type deal:

You go down, you pay your club membership and you go in there and everything's laid out on a silver platter for you. [Clive]

*Access to indecent images of children.* Group involvement has clear benefits where the distribution of indecent images is concerned, enabling offenders to amass a large and varied collection:

I was amazed at the amount of images he had … Twenty thousand plus. [Les]

This is particularly true with advancements in technology that enable the rapid sharing of files and their easy, convenient, discrete electronic storage. In an account that recalls the frenzied buzz of a bank's trading floor, Les described his shock at the scale and speed at which material was shared in online trading rooms:

I couldn't believe it the very first time I went on to it. It moved so fast, there were so many people in there, so many people talking at once and advertising and trading. [Les]

According to Les, online CSA groups involved in image-exchange particularly value two types of offenders—first, those collectors with extensive, well-labelled collections including rare series, rather than messy and ubiquitous material:

You can tell if someone's got real crap: they've got images like 1, 2, 3, 4, x, y, z. Instead of series names like Angela 1–10. [Les]

Secondly, producers are valued, as their involvement in a group guarantees brand-new and exclusive content. The first time Les posted his own images online it was to one co-offender alone, who promised to circulate them no further. Trust can be a fragile commodity, however, and this contact leaked the images to his other groups:

I'd actually given some to him on the condition that he never spread them and [it] was my naivety to say, ‘These don't go anywhere, there are personal they never get traded. [Les]

While increased access to indecent images is a desirable commodity, it carries heightened risk. Simon was jailed after his “treasure-trove” of indecent material was discovered by the police. This “Aladdin's Cave”, as he called it, of hard-copy images was an amalgam of his own collection and that of his co-offender: the latter clearly benefited from his group involvement because he could leave his precious collection for safeguarding while out of the country. Both Les and Simon emphasised the risk in disposing of hard-copy CSA material in particular:

I'd always meant and wanted to try to sort it out but what do you do with the damn stuff? You can't go and take it down to the tip. [Simon]

I didn't want to just throw it in the bin for someone to find it. I didn't want to just go and burn it in case someone saw what I was doing. [Les]

*Access to specialist skills.* Joining a group can give offenders access to others with specialist skills useful in enabling offending and evading detection. Such skills may be put to the group's services or taught actively to members: the latter is more dangerous than the former, as it protects against a single point of vulnerability and increases capability and resilience. When Simon started taking indecent photographs of children, amateurs could typically only develop their creations in black and white. One of his groups included a professional photographer, whose ability to develop in colour made him valuable to the group. From a skill perspective, however, group involvement carried most obvious benefits to Les when he became involved in online CSA. He selected a username to emphasise his “newbie” status and elicit sympathy and guidance. This worked, and Les quickly became involved in a tightly knit group of four, including one offender with considerable technological abilities: he taught the other three techniques to share material rapidly and effectively and protect themselves against detection. Consequently, Les became technologically literate very quickly, at a time when few people were:

I've just learned by other people telling me what to do. Yes, I'm an intelligent bloke, I learn quickly, but I'm not a master computer engineer. [Les]

*Access to psychological rewards.* Alongside the practical, tangible benefits detailed above, group involvement can bring considerable psychological gains. Awareness of others’ offending helped neutralise Simon's own qualms, spurring him on to experiment with intrusive forms of abuse against “well-worn”, and therefore in his eyes legitimate, victims. Moreover, group involvement offered some clear relief after years of concealing their highly taboo sexual interests. Simon compared his groups to Alcoholics Anonymous, offering:

Mutual society, help and comfort … in prosperity and adversity. [Simon]

For Les, the expressive function of his online groups soon overtook even the instrumental one of collecting images:

Towards the end my preoccupation was community-based, rather than sexually based. I dropped everything for the fact I was in an online community of people I could talk to, that understood me, that I could talk openly and honestly to. [Les]

He would leave work mid-shift to go home and go online and eventually began to socialise with his online group contacts offline as well. His descriptions of their meetings recall a small-scale sex offender convention, whereby alongside discussing shared deviant interests in his home they went drinking in the pub, a form of more conventional male bonding. Although emphatic that his stepchildren were off limits, the meetings enabled him to show off his victims:

For them, from what I understand, it was like meeting a star, if you like. Somebody that they'd seen doing things with me and to actually see in real life. [Les]

### Evolution of the group

There was evidence in these data of four ways in which groups evolved, varying in speed and deliberateness, and a fifth is also posited.

*Expansion.* A group can expand organically or through the purposeful introduction or recruitment of new members, for which the processes involved have already been discussed. According to Clive, “everything works in a circle, it all starts with one man in the middle”. As expansion can be risky, groups may implement protective mechanisms, some of which were described by Les in relation to online CSA:

He wouldn't tell me the name or any of the details, just the fact that this secret channel existed … That's when I went into the vetting procedure. [Les]

First of all you'd trade with them … on the understanding that law enforcement couldn't trade … and once you've traded with them successfully and without any hassle … you'd pull them into a personal chat and say, ‘Would you like to join an exclusive channel?’ [Les]

*Depletion.* Groups may deplete for numerous reasons, including arrests, members’ relocations or deteriorating group dynamics arising, for example, through breach of trust or internal rivalry. Despite stereotypes of a cohesive CSA group in which shared sexual deviance outweighs all differences, from these data it was clear that members may have little in common. There was extensive evidence of tensions and disputes, as well as gradual drifting apart:

[I] cut myself off from the people in that association now because I reckon most are a little bit too dodgy. [Simon]

He tried to pick up a [colleague's] son one day. What does that make me look like? A bloody idiot. [Clive]

That was the first inkling … [that] absolutely everything had to be done his way. [Simon]

*Schism.* Schisms occur when a group fragments into two or more groups. Les described a dramatic schism creating two rival groups from a notorious online collective:

They'd split … they had an argument over security … and there were personality problems and they got upset with each other. [Les]

*Cessation.* A group may simply cease to exist, temporarily or permanently, for example if an entire group is arrested or simply as the natural conclusion of a gradual depletion:

We fell out completely. [Simon]

I stayed offline after Kidster was arrested, because I'd been trading with him the night before his arrest and I panicked. [Les]

It can be difficult to establish if a group has ceased to function, relocated or simply paused its activities temporarily. Moreover, as offenders may be involved in multiple groups, a group's cessation need not signal the end of its members’ activities.

*Merger.* Although there was no evidence of this process in this data set, the reverse counterpart to a schism could be a merger: the unification of two or more groups into a single group. Indeed, police evidence suggested that the two groups formed by the schism described above subsequently reformed in this manner.

## Discussion

The powerful influence of groups on their members’ thoughts and actions has been well documented, although much situational research has overlooked the immediate social drivers of crime in favour of physical drivers (Andreson & Felson, 2010b). The premise for this study, therefore, was that involvement in a CSA group constituted a social situation in itself with a direct influence on offending behaviour. Through the systematic analysis of in-depth behavioural data the complex, multifaceted phenomenon of the CSA group could be deconstructed to identify certain processes and factors underpinning its form and function. A non-exhaustive set of four key themes, each with corresponding subthemes, were thus identified, relating to modes of formation, (self-)identification, interpersonal exchange and evolution. It is suggested that it should be possible to assess most, if not all, CSA groups against these overarching themes. The results derive from extensive interviews with three subjects, although each was involved in multiple CSA groups: this limited sample constrains the findings’ generalisability. Moreover, it should be emphasised that information was filtered through these individuals’ perspectives, as is inevitable in interview-based methods. This study's findings are neither exhaustive nor conclusive. Rather, they comprise initial and tentative steps towards establishing a unified framework for the situational analysis of CSA groups that can be used to inform targeted countermeasures.

What differentiates this study from previous investigations of CSA groups, and indeed of other crime groups, is its attempt to establish a preliminary framework for assessing such groups. An encouraging indication of the framework's broad applicability is how it aligns with findings from earlier studies that described particular characteristics of specific CSA groups. For example, psychological rewards derived from involvement in CSA forums have been an important theme in research into online CSA (Durkin, 1997; Durkin & Bryant, 1999; Durkin, Forsyth, & Quinn, 2006; Quayle & Taylor, 2002; Taylor & Quayle, 2003). Meanwhile, the largest offline CSE groups in the United Kingdom to date formed from pre-existing social networks (Cockbain, Brayley, & Laycock, 2011), a key pathway to involvement noted here and one that challenges the stereotype of CSA groups formed around shared sexual deviance alone.

Commonalities was also identified with crime research more broadly. The range of tangible and intangible commodities provided by group involvement, for example, recalls Ekblom and Tilley's (2000) situational work on resources for crime. In fact, the CSA group, in their terms a “collaborative resource”, was found here to be a source in itself of other categories they propose, namely faciliatory, cognitive and moral resources. Although commercial CSA was not the norm for these subjects, their discussions of syndicates and group discounts were a timely reminder of a business model approach, something associated more often with sex trafficking (Shelley, 2003) than CSA. The wide range of resources available through group involvement may explain why individuals remain in CSA groups despite the elevated actual or perceived risk. Here it is worth noting that the “differential apprehension hazard” theory (Erickson, 1971), which posits that co-offenders run a greater risk of detection than solo offenders, has found little empirical support (Feyerherm, 1980).

While comparative analysis of subjects’ solo and co-offending was beyond the remit of this preliminary study, it should be noted that all three subjects, chosen for their group involvement, also committed solo offences. Moreover, they were all involved in multiple, often concurrent groups, whose duration, stability and other characteristics varied greatly. Such variation challenged the notion of CSA groups as self-explanatory units, in which shared deviance trumps or obscures any distinctions between members. Together, the results underline the complex, heterogeneous and dynamic nature of CSA groups, which corresponds with findings from the co-offending and organised crime literatures (Hindelang, 1971, 1976; Paoli, 2002; Reiss, 1988; Weerman, 2003). This is a strong argument against treating CSA groups as a “special case”: much may be learned through greater integration with the broader literatures on group involvement in crime.

The results of this small-scale, exploratory investigation need to be corroborated and further developed through both qualitative and quantitative studies. Qualitative analysis of a much larger sample, for example, could test the applicability of this framework's existing components and identify missing themes and subthemes. Quantitative analysis, meanwhile, could test for the effects of variables such as CSA subtype, members’ gender or group size on the type of subtheme manifested.

Traditional responses based on lone offenders may be insufficient and ill-guided when dealing with groups (Reiss, 1988). Effective preventative responses to CSA groups require a clear appreciation of the situational processes underpinning their activity and associations. A better understanding of how such groups form and function could help inform nuanced, evidence-led responses, both in terms of counterstrategies aimed at particular types of CSA groups and tactical responses to specific target groups. This study provides the early foundations of a framework for assessing and responding to CSA groups in a systematic, transparent and replicable manner. The application and further development of these early findings could help to build a strong, coherent literature around one of the most neglected elements to the complex social issue that is CSA. As one of the greatest social influences on individual behaviour, the group's role commands greater attention.
